# On the Commemorative Ceremony and Symposium for the 20^th^ Anniversary of Food Safety Commission, Cabinet Office, Government of Japan

**DOI:** 10.14252/foodsafetyfscj.D-24-00005

**Published:** 2024-03-22

**Authors:** Shigeki Yamamoto, Satoshi Asano, Toru Kawanishi, Masako Waki

**Affiliations:** Food Safety Commission, Cabinet Office, Government of Japan, Akasaka Park Bldg 22F, 5-2-20 Akasaka, Minato-ku, Tokyo 107-6122, Japan.

**Keywords:** collaboration of risk assessment agencies, digital transformation, integrated approaches to testing and assessment, new approach methodologies, novel foods including cultured meats, sustainable development goals

## Abstract

Food Safety Commission, Cabinet Office, Government of Japan (FSCJ) was established in 2003 and marked its 20^th^ anniversary in 2023. FSCJ held a commemorative ceremony and symposium to celebrate its 20^th^ anniversary at Mita conference hall (Mita, Minato-ku, Tokyo) on September 1st, 2023, which attracted a total of 164 on-site attendees including six media companies, as well as 460 online viewers. FSCJ Chairperson Dr. YAMAMOTO gave a summary of each session; Session 1 outlined the various future challenges against which risk assessment organizations must prepare. In Session 2, panelists shared information on the development of new evaluation methodologies and international collaborations in order to meet various global demands and issues. In Session 3, the FSCJ introduced its future initiatives and called for international collaboration in sharing information and expertise to address data gaps and emerging issues, to which all panelists expressed their support. The importance of personnel development to tackle these challenges was also raised. In concluding the seminar, Dr. YAMAMOTO expressed that the common understanding gained from this occasion was the most fruitful achievement, owing to the international colleagues who shared their thought-provoking presentations and insights.

## 1. Introduction

Food Safety Commission, Cabinet Office, Government of Japan (FSCJ) was established in 2003 and marked its 20^th^ anniversary in 2023. To commemorate this milestone and to discuss various emerging challenges, such as novel foods including cultured meats and new assessment methodologies, FSCJ invited distinguished experts from the European Food Safety Authority (EFSA), the U.S. Food and Drug Administration (FDA), the Organisation for Economic Co-operation and Development (OECD), Singapore Food Agency (SFA), and Chemicals Evaluation and Research Institute, Japan (CERI) to share their latest experiences.

FSCJ held a commemorative ceremony and symposium to celebrate its 20^th^ anniversary at Mita conference hall (Mita, Minato-ku, Tokyo) on September 1st, 2023, which attracted a total of 164 on-site attendees including six media companies, as well as 460 online viewers.

The outline of each segment is as below.

## 2. Program

• Commemorative ceremony for the 20^th^ anniversary of FSCJ

• The 20^th^ Anniversary Symposium “Enhancing readiness for future challenges facing risk assessment agencies”

Session 1: Future challenges facing risk assessment agencies

Session 2: Introducing new assessment methods

Session 3: Enhancing readiness for future challenges facing risk assessment agencies

## 3. Summary of the event

### 3.1 Commemorative ceremony

Following the opening remarks by Dr. YAMAMOTO Shigeki, Chairperson of FSCJ, Mr. KONO Taro, Minister of State for Consumer Affairs and Food Safety, gave a welcome address. Congratulatory speeches were made by guests of honors Ms. ANAN Hisa, Representative Director of the Association to Create a Society with Consumer Citizenship, Dr. Carlos Gonçalo das Neves, Chief Scientist of the Executive Director Office, European Food Safety Authority (EFSA), Dr. Tan Lee Kim, Director-General/Food Administration and Deputy Chief Executive Officer of Singapore Food Agency (SFA), and Mr. YODA Gaku, Deputy Director-General of the Consumer Affairs Agency.

### 3.2 Symposium

Summary of FSCJ Chairperson Dr. YAMAMOTO’s opening remark

• Sustainable Development Goals (SDGs) initiatives have become ever more relevant on a global scale in recent years, in which securing sustainable food supply systems and improving animal welfare are addressed as major agendas.

• The symposium was made up of three sessions:

Session 1: Evaluating the safety of novel foods without any history of human consumption, such as so-called “cultured meats” and foods produced using new technologies

Session 2: Exploring new methodologies and tools for risk assessments and identifying yet-to-be-elucidated novel approaches

Session 3: Based on what was discussed in the prior two sessions, exploring new approaches by risk assessment organizations, including capacity building, directions in which to take action, and potential international collaboration

## 4. Session 1: Future challenges facing risk assessment agencies

Dr. WAKI Masako, coordinator of Session 1, gave an introductory remark on the current situation.

Within the past couple of years, novel foods for which there is no history of human consumption, as well as foods produced by emerging new technologies are gradually being distributed by industries. The purpose of the session was to share experiences among risk assessment organizations in dealing with these challenges, i.e., the nature of emerging risks in relation to innovations, and the methods to predict and ensure readiness against these risks.

### 4.1 Summary of presentation 1: “Singapore’s regulatory framework for novel foods”


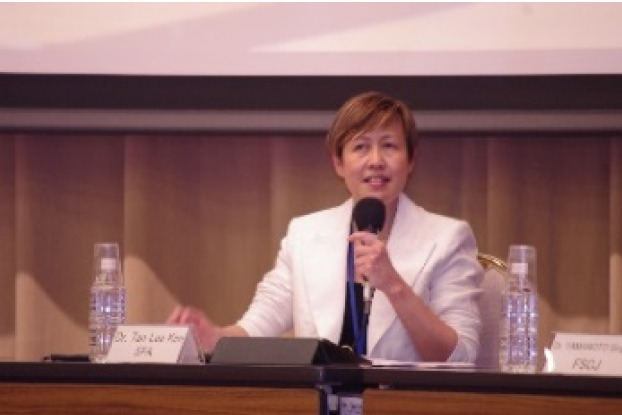


Dr. Tan Lee Kim, Director-General/Food Administration and Deputy Chief Executive Officer, SFA, introduced regulatory pathways on novel food in Singapore, where over 90% of its food supply is reliant on import. An anticipatory stance is taken on novel food with principal considerations on science-based food safety to protect consumers while preventing the stifling of novel food innovations. This stance taken by SFA allows to address global food security challenges. The regulatory approval steps of the world’s first cell-based meat (chicken) were part of SFA’s measures to identify and control risks related to pre-market novel food; 1. Pre-market safety assessment by company; SFA’s regulations require companies to identify risks related to all raw materials (e.g., cell lines, culture media, scaffold material), manufacturing process, and final products. Companies must also demonstrate that appropriate risk mitigations have been implemented. Data and results of the assessment are submitted for SFA’s review. 2. Review by SFA’s experts; The submitted data are reviewed rigorously in line with standards. 3. Compliance with standards; The product must also comply with the chemical, microbiological and labelling requirements specified under Singapore’s Food Regulations, just like any other food for sale in Singapore. 4. Licensing of local food production facilities; To ensure safety of novel food, a detailed breakdown is required upon licensing. 5. Market surveillance; Surveillance is conducted to detect any potential hazards for risk assessment, and 6. Risk communication activities take place to address perceived risks of novel foods.

The current regulatory approach of case-by-case risk assessment will soon be outpaced by the dynamic novel food ecosystem. Issues include lack of internationally harmonized guidelines and standards on risk assessment, regulation of production facilities, and labeling. Risk communication challenges include addressing consumers susceptible to misinformation. Technical challenges include methodologies to assess the risk of undesirable substances in cell-based meat and cell-based seafood. This could be due to genome instability and consequential genetic drift. Verification of novel food is also crucial to ensure that the product being produced is the version that SFA approved. The SFA is deepening its knowledge on the fundamental science of novel foods, and strongly encourages international conversations on safety assessment and regulation of novel food. The SFA hopes to establish internationally harmonized guidelines and standards, which are necessary for ensuring the safety of novel foods and facilitating trade.

Lastly, besides cell-based chicken, Dr. Kim also touched upon other novel foods approved by the SFA through its regulatory framework, including beta-lactoglobulin produced by precision fermentation.

### 4.2 Summary of presentation 2: “FDA assessment approaches for innovative foods and food ingredients”


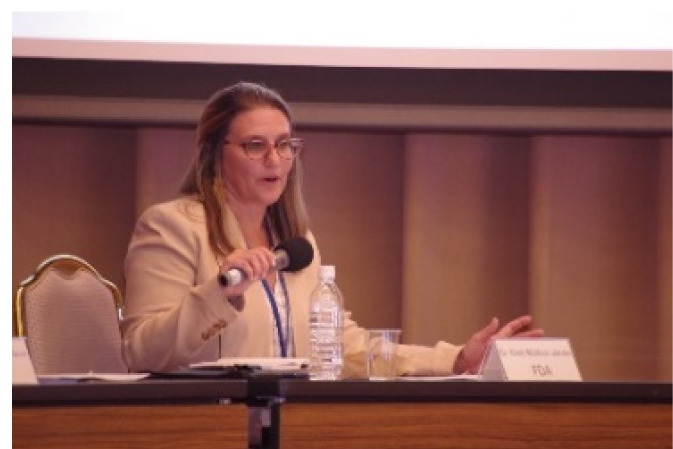


Dr. Kristi L. Muldoon Jacobs, Director, Office of Food Additive Safety, Center for Food Safety and Applied Nutrition, U.S. Food and Drug Administration (FDA), introduced FDA’s primary programs for food additive and color additive petitions, Generally Recognized as Safe (GRAS) and food contact substance notifications, as well as pre-market programs that support food innovation, including those for nanoscale substances produced by modern biotechnology. All of the above involve the expertise of an array of scientists under the tenets of the Federal Food, Drug, and Cosmetic Act, which prescribes the standard of safety and specific definitions of terms including “food additives” and “adulterated food.”

FDA’s basic standpoint on food safety assessment for substances intentionally added to food was also explained. This includes coming up with exposure estimates among consumers based on the maximum intended use levels, and evaluating whether the exposure is within safe levels. The safety of novel foods relies heavily on manufacturing process. Risks are generally examined in a case-by-case approach based on their properties, compositions, and impurities/contaminants. Since upstream components used in manufacturing of novel foods may carry through to the final food, the FDA places importance on the full understanding of manufacturing processes. Moreover, process is considered only insofar as it affects the properties of the safety of the food ingredient itself. So, it is important to understand the potential impacts of the properties that are relevant for safety, and to look at information needed to establish safety, which may change according to the manufacturing process. Human food products incorporating cultured cells from livestock and poultry was explained as a unique space in which the FDA and the US Department of Agriculture, Food Safety and Inspection Service (FSIS) jointly oversee, each sharing different responsibilities, where in this role, the FDA oversees cell collecting and culturing, and conducts pre-market consultation on the productive process. As for human foods incorporating cultured fish and seafood cells, the FDA oversees both cell culture and food processing, packaging, and labeling. Two pre-market consultations for cultured animal cell foods have been completed so far.

### 4.3 Summary of presentation 3: “Preparedness at the core of EFSA – The environmental scanning process” and “EFSA emerging risks analysis activities”


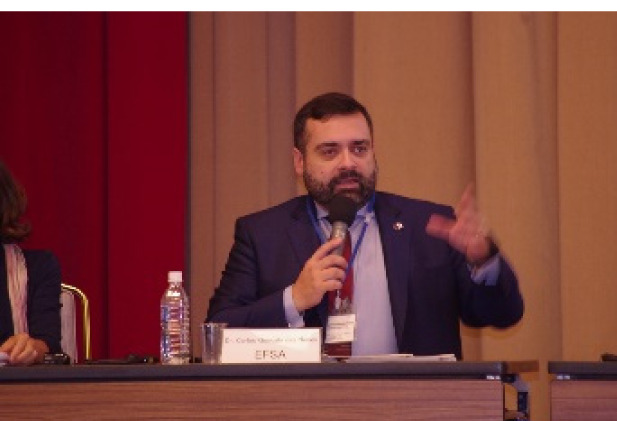


Dr. Carlos Gonçalo das Neves, Chief Scientist, Executive Director Office, European Food Safety Authority, explained how EFSA’s strategic approaches prepare for food-related affairs in the future, which are being affected by the rapidly changing aspects surrounding social, economic, and the global environmental ecosystem including global climate change, land use change, biodiversity loss, depletion of natural resources, pandemics, war, and social unrest. Amid such increased complexity, food safety assessment requires the best science, transparency, inclusivity, and speed. It is easier said than done, since it requires data sharing, scientific expertise, and transdisciplinary approaches to overcome current barriers. Specialized units were introduced under EFSA Strategy 2027 to identify emerging risks upon conducting environmental scanning/foresight.


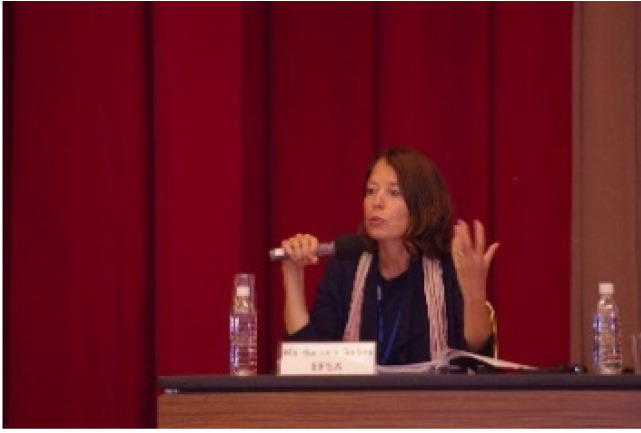


Following Dr. das Neves’ presentation, Ms. Barbara Gallani, Head of Communication and Partnership Department, EFSA, explained further on EFSA’s mission. Based on the food law in Europe (Articles 23 & 24, Regulation (EC)178/2002), EFSA has the responsibility to identify and characterize emerging risks and to establish monitoring procedures in search of ‘signals,’ whereby a ‘network of networks’ is needed in the face of complexity. An example of existing network is the Interactive Rapid Alert System for Food and Feed (iRASFF) established in Europe in which participating countries are able to access certain aspects of emerging risks. There are two other central networks; 1. The Stakeholder Discussion Group on Emerging Risks (STADG-ER), a thematic discussion group in which stakeholders from consumer organizations, industry trade associations, and veterinarian associations convene regularly to exchange specialist knowledge, and 2. Emerging Risks Exchange Network (EREN), an international liaison group encompassing EU Member States, observer countries, and international organizations including FAO/WHO. Signals collated by these two networks are screened and sorted to consider whether mandates should be given to EFSA or others for proper risk assessment. Observing the trends in emerging risks between 2015 and 2023, most signals captured were around chemical hazards until 2017, after which they were mostly microbiological hazards, as well as new consumer trends including different patterns of consumption and food substitutions. It is important to discuss at an international level on how to identify emerging risks in foresight.

During the panel discussion coordinated by Dr. WAKI, opinions were exchanged on the following topics:

• How to manage the task of identifying hazards and how to establish the assessment methods for risk assessment of novel foods, such as so-called cultured meats

• The time required from pre-market consultations for manufacturers to approval of products

• Experiences in risk communication to consumers and business sectors regarding novel foods, and points to be considered in said communication

• Key points in prioritizing various potential issues to be tackled in the future

• The implications of changing consumer trends on food safety

Through Session 1, participants reaffirmed the importance of reinforcing international cooperation to identify and address challenges in novel food safety issues.

## 5. Session 2: Introducing new assessment methods

Dr. KAWANISHI Toru, coordinator of Session 2, introduced the recent global discussions on New Approach Methodologies (NAMs) in evaluating the risks of chemical substances such as pesticide residues, food additives and pollutants. These discussions deal with animal welfare and limitations to predict human toxicity in conventional animal testing methods due to species differences between animals and humans. The following three presentations were given as examples of initiatives carried out to this end.

### 5.1 Summary of presentation 1: “Gaining confidence in the use of New Approach Methods through Integrated Approaches to Testing and Assessment”


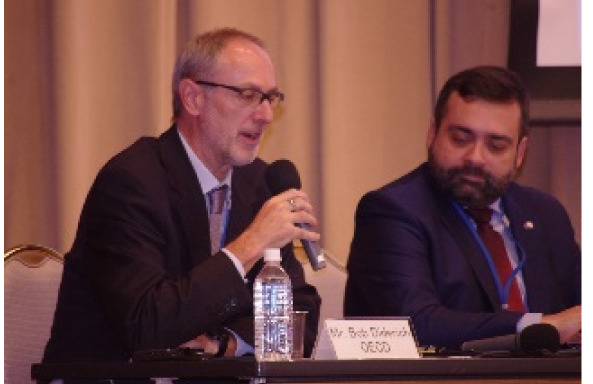


Mr. Bob Diderich, Head of the Environment, Health and Safety Division, Organisation for Economic Co-operation and Development (OECD), explained that OECD has developed a series of test guidelines, since many chemical substances on the market have not been evaluated for safety. These test guidelines and mutual acceptance of data would allow OECD member countries to harmonize test methods, which reduce the otherwise redundant testing time and costs incurred among multiple countries. Main projects on OECD Test Guidelines Programs today are about harmonization of non-animal methods. An example of non-animal test method recently published is the harmonized guideline for the evaluation of skin sensitization. One very prominent on-going project is development of *in vitro *battery assays to predict developmental neurotoxicity of chemicals fully replacing animal studies.

OECD supports the use of New approach methods (NAMs) when their suitability can be demonstrated. The working definition of NAMs in OECD basically applies to any approaches including *in chemico, in vitro, in silico,* and *in vivo* methods that are not “old”. NAMs can be stand-alone or (more often) integrated approaches to testing and assessment (IATAs). However, the hurdle for a NAM to become an OECD test guideline is quite high, since it needs to be as good as or better than existing approaches, applicable to various chemical substances, and the results need to be reproducible.

IATAs combine multiple sources of information, which include existing information from scientific literature or other resources, along with newly generated data resulting from new methods or traditional toxicity testing methods to fill data gaps and to draw conclusions on the toxicity of chemicals. These approaches are developed to address a specific regulatory scenario or for decision-making.

Formulation of IATAs is currently progressing and being tested by OECD in on-going IATA case study projects. The first wave of NAMs that have been reviewed as part of the OECD IATA case studies project were pathway-defined NAMs with good understanding of the mechanism of action aligning with Adverse Outcome Pathway (AOP). OECD predicts that the next wave of NAMs will be pathway-undefined NAMs, meaning test systems that mimic (human) biology. Currently, IATA case study projects are progressing, and member countries are sharing experiences in evaluation. OECD is trying to provide guidance for regulations based on the lessons learned in the reviews. In conclusion, Mr. Diderich expressed that OECD is looking forward to Japan’s continued rapport to drive the harmonization of NAMs.

### 5.2 Summary of presentation 2: “Implementing New Approach Methodologies: challenges & opportunities – Food & feed regulatory context”

Dr. Carlos Gonçalo das Neves, Chief Scientist and Executive Director, EFSA, provided the background on EFSA’s high priority given to the introduction of New Approach Methodologies (NAMs), which was partially in response to the strong demand from EC citizens to promote the 3Rs (Replacement, Reduction, and Refinement) of animal testing.

EFSA has an ambitious goal to routinely use NAMs to address data gaps by around 2030 and phase out animal testing. There are currently 32 ongoing projects in EFSA around the topics of NAMs that focus on areas including toxicokinetics, toxicodynamics, and read-across. In the near future, Europe will start to phase out traditional approaches and phase in data driven approaches using NAMs. EFSA’s ambition is to integrate exposome data with NAMs for exposure assessment.

Dr. das Neves also highlighted the knowledge and innovation community to build up the capacity in NAMs and touched upon his hopes for further collaboration with FSCJ.

### 5.3 Summary of presentation 3: “Promotion and issues of the NAMs approach in food risk assessment in Japan”


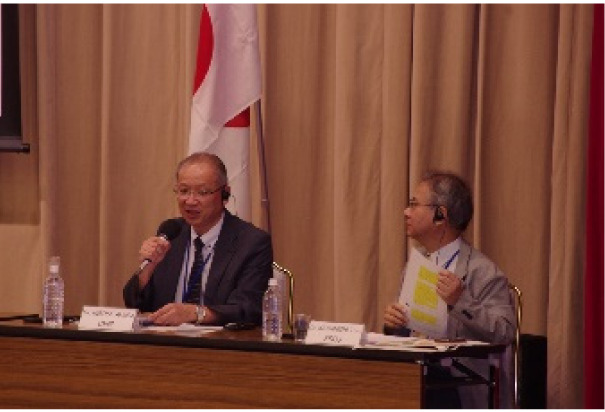


Dr. HIROSE Akihiko, Science Advisor, Chemicals Evaluation and Research Institute, Japan (CERI), explained that Japan had already been incorporating New Approach Methodologies (NAMs) in certain areas including risk assessment of food additives. The IATA framework in accordance with OECD guidelines has been used to evaluate allergenicity. For processing aids, tiered evaluation is adopted based on the Threshold of Toxicological Concern (TTC) approach, where certain toxicity testing is omitted according to estimated intake level. For flavors, (Quantitative) Structure-Activity Relationship is applied to genotoxicity evaluation, as well as TTC-based tiered assessment. Additionally, Dr. Hirose emphasized the importance of collecting data on mechanism of toxicity through international collaboration to drive NAMs-based food safety assessment.

During the panel discussion coordinated by Dr. KAWANISHI, opinions were shared, including on the following topics:

• Approaches to introduce NAMs in repeated dose toxicity study

• Potential to further integrate the concept of exposome in the assessment of various types of chemical substances

Lastly, it was reaffirmed through the session that upon risk assessments of various substances in food, NAMs and IATA are not only alternatives to animal testing, but also designed to fill in the data gaps and improve precision of evaluations using existing animal data. It was also shared among panelists that collaboration on a global scale is essential in order for NAMs to become substantial.

## 6. Session 3: Enhancing readiness for future challenges facing risk assessment agencies

Based on discussions in the previous two sessions, Dr. ASANO Satoshi, who coordinated Session 3, expressed that the aim of the session was to explore how risk assessment organizations should prepare for the future, after which the following presentation was made.

### 6.1 Summary of presentation 1: “Enhancing the capacity of FSCJ to be ready for future challenges”


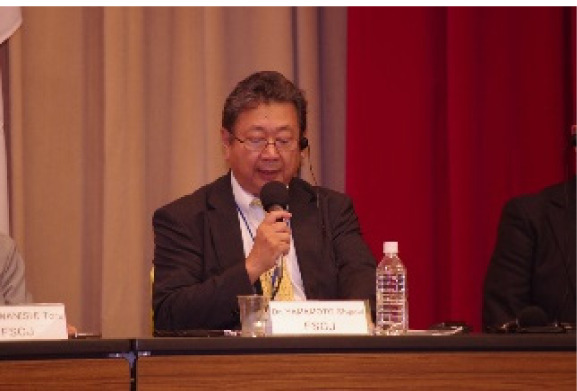


Dr. YAMAMOTO Shigeki, Chairperson, FSCJ, stressed the crucial importance of addressing future challenges including streamlining operations, transfer of the resulting surplus resources into creative work in new fields, and to boost the number of personnel taking on these creative new roles. Dr. YAMAMOTO also explained FSCJ’s current attempts to introduce digital transformation (DX) into documentation of assessment reports. This enables automatic accumulation of a vast amount of data and outcome of various deliberations in the form of a reusable real-time database. The database will be used to improve the accuracy and efficiency of safety evaluations which will result in increasing the competence of expert committee members and other risk evaluators. We will face new subjects for which assessment methodologies have not yet been established and/or address the current insufficiency of scientific information. While securing resources to tackle such new issues through these initiatives, efficient and appropriate assessment of emerging food-related risks would be possible under collaboration and sharing information with international risk assessment organizations. On behalf of FSCJ, Dr. YAMAMOTO expressed his hopes to further reinforce international ties going forward, to increase the sharing of information on risk assessments and risk communications, as well as to exchange experience in human resource development and basic mindsets for risk communications.

During the discussion coordinated by Dr. ASANO Satoshi, the following opinions were expressed by the distinguished panelists:

• Ms. Gallani, EFSA: “From a European perspective, cooperation on a global level is central to the future of risk assessment and risk communication for consumer protection. EFSA engages with various stakeholders both in and beyond Europe. EFSA is also proud of the history of our ties with FSCJ, which dates back to the signing of the memorandum of cooperation in 2009. On behalf of EFSA, I congratulate you on your 20^th^ anniversary and look forward to many more opportunities to work together, with organizations worldwide to create a more conclusive environmental risk assessment.”

• Dr. Kim, SFA: “I share Dr. YAMAMOTO’s view on the challenges we have and strongly support FSCJ’s call for international collaborations to strengthen our risk assessment capabilities and on risk communications. I would like to add that firstly, it may be useful to share each agency’s insight into new emerging areas on which we are working. Secondly, given that we all face resource constraints, we could look at how to conduct some joint risk assessment in those areas, especially in novel food. Thirdly, it may be ideal to also have a community of practice for international collaboration to build relationships, apart from science, where we could casually reach out to each other to share stories.”

• Mr. Diderich, OECD: “FSCJ’s suggestion that the IATA OECD case study project could be a model for international cooperation in food safety is a great suggestion. Nevertheless, the ultimate goal of international cooperation on risk assessment is the harmonization of risk assessment methodologies, so that the results obtained in one country on an emerging issue can be reused in another country. The harmonization can reduce the resources needed to ensure the safety of chemicals or of novel foods and feeds, since ensuring the safety of chemicals and novel foods and feeds is incredibly expensive. While IATAs are excellent tools, they are just a step towards this ultimate goal.”

• Dr. Muldoon Jacobs, FDA: “We are clearly living in an information age. Creating systems that can digitally leverage data that has been collected previously with current and future data, in enough format, would increase efficiencies and the reliability of decision making. As Dr. YAMAMOTO noted, it is clear that cooperation, collaboration, and communication will absolutely be essential to achieve this. FDA is committed to transparency and information sharing that relates to food safety for the benefit of all local consumers and regulators and we share that vision. Better data integration and sharing can help leverage all of the global resources to those seeking data information that supports the safety and the risk assessments going forward. I look forward to building on a new relationship that has been set here today.”

• Dr. HIROSE, CERI: “While it has often been said that harmonization is important, details are yet to be discussed. This symposium had served as a valuable opportunity for the four organizations to come together in reaffirming the shared international interest toward harmonization. It may be ideal to collaborate on a few OECD case study projects also, as an example of a concrete plan in the future.”

• Dr. WAKI, FSCJ: “It was shared among international colleagues that each international region has been working under limited resources, and that strengthening mutual trust among international organizations is important to proceed with substantive discussions. This symposium was a good opportunity to build international rapport and to further unite for a common purpose.”

• Dr. MATSUNAGA, FSCJ: “The importance of effective risk communication toward the public was emphasized. EFSA’s presentation on food fraud and changing consumer perspectives that are bringing about emerging risks have left a strong impression. How to communicate to the general public about the collaboration efforts among relevant agencies and to foster trust in risk analysis was also touched upon, which was thought-provoking. I would also like to continue dialogue with our international colleagues, deepen our communication, and strengthen our mutual understanding.”

FSCJ Chairperson Dr. YAMAMOTO gave a summary of each session; Session 1 outlined the various future challenges against which risk assessment organizations must prepare. In Session 2, panelists shared information on the development of new evaluation methodologies and international collaborations in order to meet various global demands and issues. In Session 3, the FSCJ introduced its future initiatives and called for international collaboration in sharing information and expertise to address data gaps and emerging issues, to which all panelists expressed their support. The importance of personnel development to tackle these challenges was also raised.

In concluding the seminar, Dr. YAMAMOTO expressed that the common understanding gained from this occasion was the most fruitful achievement, owing to the international colleagues who shared their thought-provoking presentations and insights.

